# femtoPro: virtual-reality interactive training simulator of an ultrafast laser laboratory

**DOI:** 10.1007/s00340-023-08018-7

**Published:** 2023-04-29

**Authors:** Tobias Brixner, Stefan Mueller, Andreas Müller, Andreas Knote, Wilhelm Schnepp, Samuel Truman, Anne Vetter, Sebastian von Mammen

**Affiliations:** 1 Institut für Physikalische und Theoretische Chemie, Universität Würzburg, Am Hubland, 97074 Würzburg, Germany; 2 Games Engineering, Institut für Informatik, Universität Würzburg, Am Hubland, 97074 Würzburg, Germany

## Abstract

The huge field of optics and photonics research and development is in constant demand of well-trained experts. However, it is challenging to teach efficiently the setup process of complicated optical experiments due to limited hardware availability and eye-safety concerns, in particular, in the case of femtosecond lasers. We have developed an interactive simulation of an ultrafast laser laboratory (“femtoPro”) for teaching and training, implementing physical models for the calculation and visualization of Gaussian laser beam propagation, ultrashort optical pulses, their modulation by typical optical elements, and linear as well as nonlinear light–matter interaction. This facilitates the setup and simulated measurement procedure, in virtual reality (VR) and at real-time speeds, of various typical optical arrangements and spectroscopy schemes such as telescopes, interferometers, or pulse characterization. femtoPro can be employed to supplement academic teaching in connection with regular courses in optics or spectroscopy, to train future scientists and engineers in the field of (ultrafast) optics in practical skills, to communicate to other researchers how to set up and align a particular experiment, to “test-build” and simulate new designs of optical setups, to simulate ultrafast spectroscopy data, to offer practical exercises to high-school students, and to reach out to the general public.

## Introduction

The optics and photonics industry is an immensely huge and commercially relevant field. According to the SPIE 2020 Optics and Photonics Industry Report [1], “annual revenues from production of optics and photonics core components amounted to 282 billion USD in 2018,” with a “compound annual growth rate (CAGR) of 10.6% for the 2016–2018 period” and “more than four million jobs worldwide” associated with the fabrication of “photonics-enabled products.” Lasers and photonics-enabled products are abundant in daily life, research, and industry. Applications can be found across all disciplines of fundamental and applied sciences and engineering. In particular, femtosecond (fs) lasers become more and more widespread for applications in biology, medicine, material processing, and advanced manufacturing. Optics-related emerging quantum technologies for metrology, sensing, communications, and computing are also developed intensively and are funded within the European Union “Quantum Flagship” program with 1 billion EUR (since 2018). Last but not least, many research groups worldwide employ ultrafast lasers for time-resolved spectroscopy.

Clearly, there is a rising demand for well-trained experts in (ultrafast) laser technologies and the corresponding need for appropriate education and training in academic and industry contexts. Theoretical aspects can be covered reasonably well with classroom-style lectures, video screencasts, books on general optics [2–7] and, in particular, on ultrafast laser pulses and the principles of time-resolved spectroscopy [8–16], or the scientific literature. A satisfying implementation of practical training, however, is much more challenging. Femtosecond laser systems that are already installed in a particular location are typically needed full time for research or production tasks and cannot be spared for education for extended time periods. While acquisition costs have dropped for fs lasers, it is still a significant financial investment to set up a complete ultrafast laboratory, surpassing the financial capacities of most educational institutions. Even if the infrastructure is available, qualified personnel needs to be available for constant supervision, both to teach specific optics skills and to ensure that laser (eye) safety protocols are followed. It is typically not feasible to offer to a large number of students or other trainees such an extended lab experience. Lastly, the COVID-19 epidemic has called for new teaching concepts that facilitate reducing direct person-to-person contacts that would occur with normal tutoring.

Computers are very helpful for simulating various aspects of optical systems, lasers, and spectroscopy experiments. For example, ray tracing [17] is used in the design process of optical elements to predict and optimize beam paths and imaging properties, and numerous software solutions exist for various purposes [18]; finite-difference methods and other “Maxwell solvers” [19–24] provide accurate details even down to the nano-optical regime [5]; and spectroscopy experiments can be simulated by treating the quantum dynamics of light–matter interaction, with several software implementations being available [25–29]. Thomas Feurer and colleagues developed a package for simulating the spectral–temporal aspects of fs experiments in a graphically intuitive manner using the LabView platform [30], which was inspiring for the present work.

While the described existing solutions all have their justified and well-established fields of application, they do not offer practical laboratory exercise. Recently, virtual-reality (VR) technology has become both affordable and mature enough for use in professional training applications. VR training domains range from dental medicine [31] over first-aid [32] to classroom management [33]. They also encompass various engineering disciplines such as construction [34], surveying [35], and electrical engineering [36]. Most relevant for the present work are those training experiences that focus on optics. In 1996, a VR laser physics laboratory was presented, tailored to pupils, giving them the opportunity to explore the dualism of light, beam propagation and wave characteristics on their own agenda [37]. In a VR training experience on fiber optics engineering, students were empowered to interactively create empirical setups to investigate characteristics of laser diodes and fiber coupling [38]. In another example, students were provided a web client to query actual solid-state pulsed lasers from remote and could also set up more complex experiments in VR [39]. Another report aims at learning to handle ultrafast lasers [40]. A recent demonstrator promises broad application of VR training in photonics and optics experimentation [41], and another VR optics lab implementation is about to enter the market [42].

As pointed out by the cited works on optics that we have come across, various advantages of a virtual, VR solution were emphasized. These include the greater safety of the students, the greater availability due to reduced costs (hardware, personnel, rooms) but also the means to display additional and didactically useful information, such as the visualization of laser beams in space and details of pulse properties at any position, which would not be available in a real-world laboratory. In addition to teaching the practical handling of general lasers and optics, accurate physical models of light–matter interaction would allow extending the scope to the simulation of spectroscopic experiments with applications in research, including in ultrafast science, which additionally motivated our work.

We have developed an interactive training software, termed “femtoPro” [43], that simulates a complete ultrafast laser laboratory on a stand-alone VR platform, taking into account the particular properties of fs pulses. The user wears VR goggles and manipulates optical elements on a VR laser table such as the coarse position and fine alignment of mirrors, lenses, irises, or delay stages. Laser-beam propagation is simulated and displayed at real time using Gaussian beams. Within this paper, we define “real time” not in the sense of inherent fs dynamics, but to signify that the simulation time for a complete spectroscopy experiment is on the order of 10 ms on a typical VR headset. In that case, frame rates of 100 Hz can be reached, ensuring a smooth user experience. Then, the user does not notice a timing difference between carrying out any linear or nonlinear optical experiment in a real laboratory or simulating the same setup in femtoPro. Interaction with matter is treated via linear and nonlinear response functions. Observables are recorded in VR spectrometers within VR data-acquisition software. In this fashion, the user can build and test, in an eye-safe manner, entire spectroscopy setups with all the required fine-tuning steps. The teaching aspect is realized with a “training mission” system that provides step-by-step guidance through alignment procedures.

In the present work, we describe the essence of the physical model underlying femtoPro (Sect. 2), relevant aspects of the simulation algorithm and code (Sect. 3), an example, training missions, and application scenarios (Sect. 4), and then we conclude (Sect. 5).

## Physical model

Setting up a mathematical model of an ultrafast laser lab, one has to balance physical accuracy and simulation cost. The various choices were dictated by the requirement to implement real-time simulation of beam propagation, pulse modification, and light–matter interaction. We describe the resulting model in the following subsections.

### Laser

Using a “Maxwell solver” would be too expensive for real-time applications. At the opposite limit of retaining only few simulation details, purely geometrical optics using “light rays” would not capture the essence of a finite laser beam radius and its evolution that form integral components in didactically relevant beam-overlap optimization procedures. Thus, we decided to base the simulation on Gaussian beam propagation [44] using a complex radius of curvature, $q\in \mathbb {C}$, that is related to the real curvature radius of the wave front, $R\in \mathbb {R}$, and the beam radius, $w\in \mathbb {R}$, according to

1 $$\begin{aligned} \frac{1}{q(z)}=\frac{1}{R(z)}+i\frac{\lambda M^2}{\pi w^2(z)} \end{aligned}$$

at a position *z* for propagation along the $\hat{\textbf{z}}$ direction, wavelength $\lambda$, and beam-quality parameter $M^2\ge 1$ (for Gaussian beams, $M^2=1$). The beam radius *w*(*z*) marks the transverse distance between the center of the beam and that point at which the electric-field amplitude has dropped, with a Gaussian function, to 1/*e* with respect to its on-axis value at position *z*. Although somewhat arbitrary, we use this value to visualize laser beams in femtoPro with an appropriate mesh if a visualization option is selected (Sect. 3.4). The beam radius can be obtained by solving Eq. (1) for *w*, yielding

2 $$\begin{aligned} w(z)=\sqrt{\frac{\lambda M^2}{\pi {{\,\textrm{Im}\,}}\left[ \frac{1}{q(z)}\right] }}, \end{aligned}$$

where ${{\,\textrm{Im}\,}}$ denotes the imaginary part of a complex number.

Beam propagation in free space is governed by [45, 46]

3 $$\begin{aligned} q(z)=q_0 + z \end{aligned}$$

for propagation by a distance of *z*, where $q_0=q(0)$. Thus, given a point in space through which the center of a laser beam passes, its *q* value at that position, and a beam direction, the further evolution of the laser beam is fixed and its visualization mesh can be calculated by application of Eqs. (2) and (3). Modification of *q* by optical elements such as lenses or mirrors will be explained in Sect. 2.2.

A laser source can be treated as a beam starting at its waist position (i.e., at the location along its propagation where the beam radius is smallest) with a given waist radius $w_0$. At the waist, Gaussian beams have planar wave fronts, i.e., infinite curvature radius $R\rightarrow \infty$. Thus, from Eqs. (1) and (3), one obtains

4 $$\begin{aligned} q_{0,\text {laser}}=-iz_{\textrm {R}} \end{aligned}$$

for a laser positioned at $z=0$, with the Rayleigh length defined as

5 $$\begin{aligned} z_{\text {R}}=\frac{\pi w_0^2}{\lambda M^2}. \end{aligned}$$

**Fig. 1:**
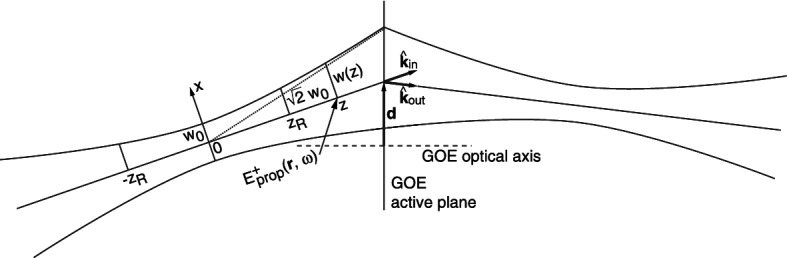
Beam geometry. The incident laser beam is on the left, the outgoing laser beam on the right of the central active plane of a general optical element (GOE) with finite focal length, e.g., a lens

At the Rayleigh length, the beam radius is higher by a factor of $\sqrt{2}$ than at the waist, and the beam radius evolution along the propagation axis follows a hyperbolic function as indicated in Fig. 1, with the asymptotic divergence angle for large distances marked as a dotted line. Note that the *z* axis defines the local axis of propagation, which in general does not coincide with the optical axis as defined by the surface normal of the optical element that is subsequently hit.

This description assumes a stigmatic beam, i.e., a radially symmetric profile. In reality, laser beams can be astigmatic. Methods have been developed in the literature to deal with astigmatic Gaussian beams, their propagation, and their modification at optical elements [47–52]. For simplicity and computational cost, however, we refrained from adopting this approach in the first implementation of femtoPro. It might still be added at a later stage.

Let us now discuss spectral–temporal laser properties. Various algorithms have been developed to simulate laser pulse propagation including linear and nonlinear effects [53]. We assume space–time coupling [54] to be absent so that we can treat the time evolution independently of beam radius and curvature. femtoPro features ultrashort pulses, and we follow for the most part the standard protocol for their description [9–12]. We transfer the continuously defined, real-valued temporal field *E*(*t*) [V/m] and the Fourier-transformed, complex-valued, spectral field $E(\omega )$ to complex-valued temporal and spectral envelopes, by removing, respectively, the steep linear temporal phase due to the fast carrier-frequency oscillation and by removing the steep linear spectral phase due to propagation in space. We also remove from the total field the change in amplitude upon propagation that arises from a change in beam radius; we treat this effect separately and explicitly. The results are stored as dimensionless arrays $\tilde{E}_t(j)$ and $\tilde{E}_\omega (j)$, sampled at a number of $N_{\text {s}}$ discrete and equidistant time and frequency positions with indices $j=\{0,1,\ldots ,N_{\text {s}}-1\}$ and sampling step sizes $\delta t$ and $\delta \omega$, respectively. From these stored arrays, we can retrieve the complex-valued positive-frequency-part temporal and spectral electric fields $E_{\text {prop}}^+(\textbf{r},t)$ and $E_{\text {prop}}^+(\textbf{r},\omega )$, respectively, that include propagation effects and spatial dependence at position $\textbf{r}$, using an appropriate scaling factor *S* that is proportional to the pulse energy and defined such that it is equal to the pulse energy for the initially emitted and $L^2$-normalized $\tilde{E}_t(j)$ with $L^2$ as the Euclidean norm.

### General optical element

When a laser pulse hits an object, it is modified. We implemented the concept of a “general optical element” (GOE) that acts upon all incident light pulses, creating modified output pulses that generally have different spatial and spectral–temporal properties. The GOE may feature intensity detection (Sect. 2.3), first-order (Sect. 2.4) or second-order (Sect. 2.5) response. Thus, we use one and the same code interface to describe linear optics encountered in a laser lab, such as screens, mirrors, lenses, or beam splitters, but also nonlinear components such as second-harmonic crystals.

Apart from the spectral–temporal modifications further described in the following subsections, we have to consider the spatial transformation and use a mix of geometrical optics and wave optics. While appropriate transformation equations for optics with finite thickness have been developed at varying degrees of accuracy, we consider only the limit of “thin optics” that deals with all beam transformations as if they occurred in one “active” plane of the optic, rather than separately at each physical interface between media. Thus, for a thin lens, we consider that an incident beam propagates in free-space until its central “ray” intersects with the GOE’s active plane, upon which the spatial transformation occurs. We calculate the new direction of a beam after the GOE using geometrical optics. Note that conventionally a coordinate system is assumed in which the paraxial approximation is applicable. In femtoPro, however, the laser beams can propagate in any direction of three-dimensional space. Thus, we need to employ a description that is independent of a specific coordinate system, with vector calculus being ideal. If $\textbf{d}$ marks the position of intersection as measured from the center of the GOE, and $\hat{\textbf{k}}_{\text {in}}$ and $\hat{\textbf{k}}_{\text {out}}$ the unit (normalized) directional incidence and output vectors, respectively, of propagation as indicated in Fig. 1, they are connected by [55]

6 $$\begin{aligned} \hat{\textbf{k}}_{\text {out}} = \frac{\hat{\textbf{k}}_{\text {in}}-\frac{\textbf{d}}{f}}{\left|\hat{\textbf{k}}_{\text {in}}-\frac{\textbf{d}}{f}\right|} \end{aligned}$$

for a GOE with focal length *f* if no beam clipping occurs. For mirrors, an additional reflection of $\hat{\textbf{k}}_{\text {out}}$ occurs at the active plane. In addition to the change in direction, the complex curvature is modified according to [45, 46]

7 $$\begin{aligned} \frac{1}{q_{\text {out}}}=\frac{1}{q_{\textrm {in}}}-\frac{1}{f}. \end{aligned}$$

Since any GOE has a finite aperture (such as the diameter of a mirror or the opening of an iris), laser beams can be clipped. This leads to diffraction that is computationally very costly to calculate and would not be feasible for a real-time simulation that has to follow many laser beams through a multitude of GOEs including light–matter interaction. Thus, we decided to ignore diffraction and implement the approximation that a clipped beam results in a new Gaussian beam whose diameter [given by twice the beam radius from Eq. (2)] is given by the common geometric cross section of the incident beam and the aperture opening. This ensures that the laser beam exiting from a GOE cannot be larger than the GOE itself. Again, we ignore astigmatism.

### Interference and detection

Interference forms an integral part of optics with coherent light. In the linear regime, the linear superposition of all incident electric fields describes the total field at any point in space and time. Thus, it is sufficient, for the treatment of free-space propagation and for the treatment of modulation at GOEs, to treat all incoming laser beams independently of each other, even if they overlap spatially and/or temporally–spectrally. We need to consider interference patterns only when evaluating nonlinear response or when measuring the total light field’s intensity, such as on a screen, in a spectrometer, or in a power meter. General-optics books usually discuss special cases of the interference pattern observed on a screen. Typically, only two beams are taken into account, they constitute planar monochromatic waves of the same frequency, and often their incidence angles fulfill certain symmetries. In femtoPro, we have to deal with the general situation of any number of incidence beams, varying complex radii of curvature, arbitrary incidence angles, relative displacements of beams, and different time and frequency structures of incident pulses.

One possibility of treating the general situation would be to calculate, explicitly, the interference pattern as a function of transverse coordinates in the detector plane, such as has been visualized for two Gaussians [56]. This would require sampling of interference fringes on a two-dimensional grid with high spatial resolution. We chose not to do so in the current implementation of femtoPro to avoid the huge computational cost. Thus, interference fringes are currently not visualized, but interference effects are taken into account for detectors in a spatially integral fashion. For fs laser pulses, the omission of spatial fringe visualization is not as severe a restriction as one might expect because the maximum path-length difference at which optical interference can be seen is given by $\Delta L=c\tau _{\text {p}}$ for a bandwidth-limited pulse duration $\tau _{\text {p}}$. Using, e.g., 10-fs pulses, one arrives at $\Delta L={3}~\upmu\textrm{m}$, and thus “most of the time” one cannot observe interference fringes anyway in an ultrafast laboratory, except very close to zero delay between pulses or if the interference is frequency-resolved, as we address now. The resulting spatially integrated and frequency-integrated interference effect is then visualized again with the brightness of the rendered beam spot (Sect. 3.4).

It is possible to describe the integral effect of interference in an effective manner when implementing a spectrometer or a power meter as detector (or when considering nonlinear optics pulse generation). In that case, we can integrate the intensity variations over the interference fringes hitting the area of the detector and simulate what a conventional spectrometer would detect. For two collinear pulses and identical spatial properties, e.g., this measurement mode is called “spectral interference” [57]. The spectral interference contrast is largest if the beam radii, beam positions, pulse energies, and directions of the incident pulses are the same. For differently sized beams, laterally displaced beams, different energies, or angular directional mismatch, the interference visibility will decrease. The analysis of such effects forms an integral part of aligning optical setups and provides relevant feedback during alignment procedures, e.g., of interferometers that are deduced to be aligned well when interference contrast is maximal. Thus, we seek an appropriate description of these effects in femtoPro and derive quantitative results for interference and contrast visibility.

**Fig. 2:**
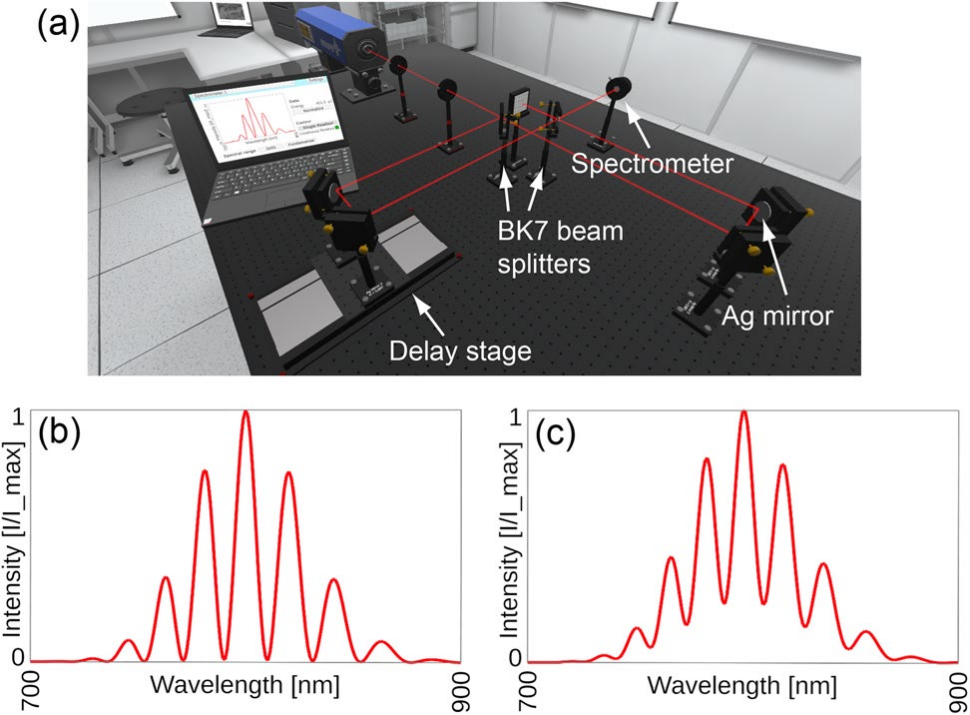
Modeling of interference. **a** Screenshot from femtoPro showing a Mach–Zehnder interferometer. **b** Spectrum of the collinear beams (pulse duration of 15 fs) in case of ideal alignment, recorded at an interpulse delay at which typical interference fringes appear. **c** Spectrum in the case of a lateral misalignment of a mirror by 2 mm. Both spectra were taken as screenshots from femtoPro

For the interference analysis, we obtain the spectral power $P_\omega$, detected by the spectrometer from an interference of *N* pulses $E_{k,\text {prop}}^+(\textbf{r},\omega )$, $k=\{1,\ldots ,N\}$, by integrating the absolute magnitude squared of the total field over transverse coordinates *x* and *y*,

8 $$\begin{aligned} P_\omega (j)&= \int _{-\infty }^{\infty }\int _{-\infty }^{\infty } \left|\sum _{k=1}^N E_{k,\text {prop}}^+(\textbf{r},\omega )\right|^2 \,\textrm{d}x\,\textrm{d}y \end{aligned}$$

9 $$\begin{aligned} =&\int _{-\infty }^{\infty }\int _{-\infty }^{\infty } \sum _{k=1}^N \left|E_{k,\text {prop}}^+(\textbf{r},\omega )\right|^2 \,\textrm{d}x\,\textrm{d}y\nonumber \\&+\int _{-\infty }^{\infty }\int _{-\infty }^{\infty } \sum _{k=2}^N\sum _{l=1}^{k-1} 2{{\,\textrm{Re}\,}}\left\{ E_{k,\text {prop}}^+(\textbf{r},\omega ) E_{l,\text {prop}}^{+*}(\textbf{r},\omega )\right\} \end{aligned}$$

10 $$\begin{aligned}&= \sum _{k=1}^N \frac{S_k}{\delta \omega }\left|\tilde{E}_{k,\omega }(j)\right|^2 \nonumber \\&+\sum _{k=2}^N\sum _{l=1}^{k-1} 2{{\,\textrm{Re}\,}}\left\{ \eta _{k,l}\frac{\sqrt{S_k S_l}}{\delta \omega } \tilde{E}_{k,\omega }(j)\tilde{E}_{l,\omega }^*(j) e^{i\omega (T_k-T_l)}\right\} , \end{aligned}$$

where the first line in Eq. (9) describes the spectral powers of the individual beams and the second line their pairwise interference, with ${{\,\textrm{Re}\,}}$ denoting the real part. The interference visibility factor $\eta _{k,l}$ in Eq. (10) results from the analysis of the spatial overlap of Gaussian functions and is found to be

11 $$\begin{aligned} \begin{aligned} \eta _{k,l}&=\frac{2w_k w_l}{w_k^2+w_l^2} \exp \left[ -\frac{(x_k-x_l)^2+(y_k-y_l)^2}{w_k^2+w_l^2}\right] \\&\times e^{i(\Delta k_x x_{\text {p}}+\Delta k_y y_{\textrm {p}})} \exp \left[ -\frac{\Delta k_x^2+\Delta k_y^2}{2\left( \frac{1}{w_k^2}+\frac{1}{w_l^2}\right) }\right] \end{aligned} \end{aligned}$$

with Gaussian beam radii $w_k$ and $w_l$, lateral beam positions $(x_k,y_k)$ and $(x_l,y_l)$, wave-vector mismatches between the two beams of $\Delta k_x$ along the $\hat{\textbf{x}}$ and $\Delta k_y$ along the $\hat{\textbf{y}}$ direction, and product beam position $(x_p,y_p)$ describing the center of the product of the two incident Gaussians, all evaluated at the position of the detector. From Eq. (10), we obtain the energy detected by a power meter via integration over frequency. Note that in Eq. (10), we use the computer-stored array representation of the complex frequency envelope, $\tilde{E}_{k,\omega }(j)$, and take into account pulse energy scaling via *S* and beam radius scaling explicitly via the visibility factor. We show the interference spectrum of two collinear superimposed beams on the example of a Mach–Zehnder interferometer (Fig. 2a) in case of ideal alignment and thus maximum interference contrast in Fig. 2b. This spectrum was recorded in VR with beam radii of $w_k = w_l = 3$ mm. The interpulse delay was set such that typical interference fringes can be seen.

The requirement for interference visibility can be used to estimate the necessary precision in optical alignment that has to be fulfilled within femtoPro and find, in agreement with the experience from a real lab where fine-adjustment and micrometer screws are employed routinely, that the lateral beam position has to be controlled to better than $\approx 0.1~\textrm{mm}$ for typical beam parameters for interference to be controlled. We showcase this circumstance in Fig. 2c, where a notably lower interferometric contrast results when the mirror indicated by the white arrow in Fig. 2a is shifted laterally by 2 mm. The interpulse delay was adjusted to produce a similar interference pattern as in Fig. 2b. Evaluating Eq. (11) with the relevant parameters, it is expected that this misalignment reduces the interference visibility to 80%, which is reflected in the observed spectrum in Fig. 2c. Thus, any (VR) alignment mechanics of GOEs should be more precise than that and reproducible, just as in a real optics laboratory. Hence, we solve this challenge with a similar approach as in the real world and separate the alignment procedure into one part of coarse positioning of elements by manually placing them in the approximately correct position and another part of fine alignment using appropriate micrometer adjustment screws. The resulting VR realization is described in Sect. 3.5. Path-length positioning should be precise to fractions of a wavelength for interferometric experiments, which is realized with delay stages that are computer-controlled.

### First-order response

We implement non-resonant first-order response that is appropriate for describing materials such as neutral-density filters, glass, or lenses (Sect. 2.4.1), and we implement resonant first-order response to describe molecular samples defined via material parameters (Sect. 2.4.2). In all cases, we multiply the frequency-domain incident field $E_{\text {in}}(\omega )$ with an appropriate modulation function $M(\omega )$,

12 $$\begin{aligned} E_{\text {out}}(\omega )=M(\omega )E_{\text {in}}(\omega ), \end{aligned}$$

to get the output field $E_{\text {out}}(\omega )$ after transmission through the material [9]. In the computer realization, we use, equivalently, the $\tilde{E}_\omega (j)$ array.

#### Non-resonant materials

Material dispersion introduces a dispersive phase such that

13 $$\begin{aligned} M_{\text {non-resonant}}(\omega )=e^{i\Phi _{\text {disp}}(\omega )}. \end{aligned}$$

One option is to employ a user-provided $\Phi _{\text {disp}}(\omega )$ that describes a specific material according to pre-characterized behavior. A second option is to perform a Taylor expansion on this phase and keep terms up to, say, third order. The relevant parameters are available for many materials and specific center wavelengths [58]. For propagation in air, we assume a group-velocity dispersion of ${20} \hbox { fs}^{2}/{\textrm{m}}$ as reported for a center wavelength of 800 nm [59]. Using Taylor coefficients instead of the fully sampled phase for dispersion treatment allows us to reduce the number of computational steps and potentially increases performance in time-critical situations. Upon transmission through dispersive materials, only the Taylor coefficients are updated, and the full phase is reconstructed numerically from the starting phase and adding the appropriate polynomials only when needed (such as when second-order response is evaluated), rather than having to numerically update the fully sampled field at each optical element.

In addition to changes of dispersive phase, it is possible to take into account spectrally uniform changes in transmission such as in neutral-density filters. For this purpose, we apply the Lambert–Beer law independent of frequency, modifying the total energy by

14 $$\begin{aligned} W_{\text {out}}=W_{\text {in}}e^{-\alpha L} \end{aligned}$$

with a given extinction coefficient $\alpha$ of the material and propagation length *L*. In practice, such a change in energy is implemented by adjusting the pulse-energy scaling factor *S*.

#### Resonant materials

For describing resonantly excited materials, we employ the response-function formalism and calculate $M_{\text {resonant}}(\omega )$ with given material parameters in a Franck–Condon model to include vibrational progression [8]. We set up the amplitude scaling of $M_{\text {resonant}}(\omega )$ such that a specified extinction coefficient $\alpha$ is fulfilled at the peak absorption according to Eq. (14). Note that we pre-calculate $M_{\text {resonant}}(\omega )$ for a given material such that the application of Eq. (12) is feasible at real-time speed, i.e., on the order of 10 ms to achieve simulation frame rates up to 100 Hz.

### Second-order response

Accurate modeling of second-order processes can be quite involved if one wants to consider realistic focusing conditions, geometric beam-overlap and beam walk-off effects, finite conversion bandwidth due to phase mismatch or group-velocity mismatch, material dispersion, birefringence of the material, saturation, and other phenomena [60–65] that may be more or less relevant depending on the particular situation. We implement some, but not all, of these effects and treat second-order processes in the limit of instantaneous (non-resonant) response such that they are most conveniently described in the temporal domain. Thus, a second-order signal field

15 $$\begin{aligned} E_{\text {s}}(t)\propto E^2(t) \end{aligned}$$

is generated. Considering two incident fields $E_1(t)$ and $E_2(t)$ with $E(t)=E_1(t)+E_2(t)$ and complex envelopes $\tilde{E}_1(t)$ and $\tilde{E}_2(t)$, this leads to the explicit terms

16 $$\begin{aligned} \begin{aligned} E^2(t)&=\tilde{E}_1^2(t)e^{i(2\textbf{k}_1\cdot \textbf{r}-2\omega _1t)}+\text {c.c.} +\tilde{E}_2^2(t)e^{i(2\textbf{k}_2\cdot \textbf{r}-2\omega _2t)}+\text {c.c.}\\&+2\tilde{E}_1(t)\tilde{E}_2(t) e^{i[(\textbf{k}_1+\textbf{k}_2)\cdot \textbf{r}-(\omega _1+\omega _2)t]}+\text {c.c.}\\&+2\tilde{E}_1(t)\tilde{E}_2^*(t) e^{i[(\textbf{k}_1-\textbf{k}_2)\cdot \textbf{r}-(\omega _1-\omega _2)t]}+\text {c.c.}\\&+ 2\left|\tilde{E}_1(t)\right|^2 + 2\left|\tilde{E}_2(t)\right|^2 \end{aligned} \end{aligned}$$

describing second-harmonic generation of pulses 1 and 2 in line 1, sum-frequency generation (SFG) in line 2, difference-frequency generation in line 3, and optical rectification of pulses 1 and 2 in line 4, with “c.c.” denoting complex conjugation of the previous term.

Note that the temporal fields as written in Eq. (16) are the “physical” electric fields, but the computer-stored complex envelopes $\tilde{E}_{k,t}(j)$ do not contain information on spatial beam-profile properties. Similar to the linear interference treatment in Sect. 2.3, we take this into account explicitly by considering the Gaussian beam overlap. In the first version of femtoPro, we include second-harmonic generation and SFG, but not difference-frequency generation and optical rectification, to remain in a limited frequency regime thus reducing the number of required spectral sampling points and improving computational speed upon Fourier transformation. But it is straightforward, in principle, to add the extension. We generate second-order fields $E_{\text {s}}(t)$ for all possible pairwise mutual combinations of incident fields. Thus, two incident fields produce two second-harmonic generation beams and one SFG beam, three incident fields produce three second-harmonic generation beams and three SFG beams, and so on (of course only if there is temporal overlap and spatial overlap).

Considering the overall energy scaling, we take into account the mutual spatial beam overlap and the individual beam radii of the incident beams. Furthermore, we ensure that the maximum energy conversion efficiency remains always below a given fraction, e.g., $10~\mathrm {\%}$, to fulfill the implicit assumption of a non-depleted three-wave-mixing process in a finite-length medium. Other than this efficiency scaling, we assume perfect phase matching but allow options to be set such that either only SFG or only second-harmonic generation or both types of second-order fields are generated for any particular nonlinear crystal. Treating phase matching in a realistic manner would require introduction of vectorial electric-field properties and birefringent materials, which we have avoided for reasons of computational cost in the present stage.

**Fig. 3:**
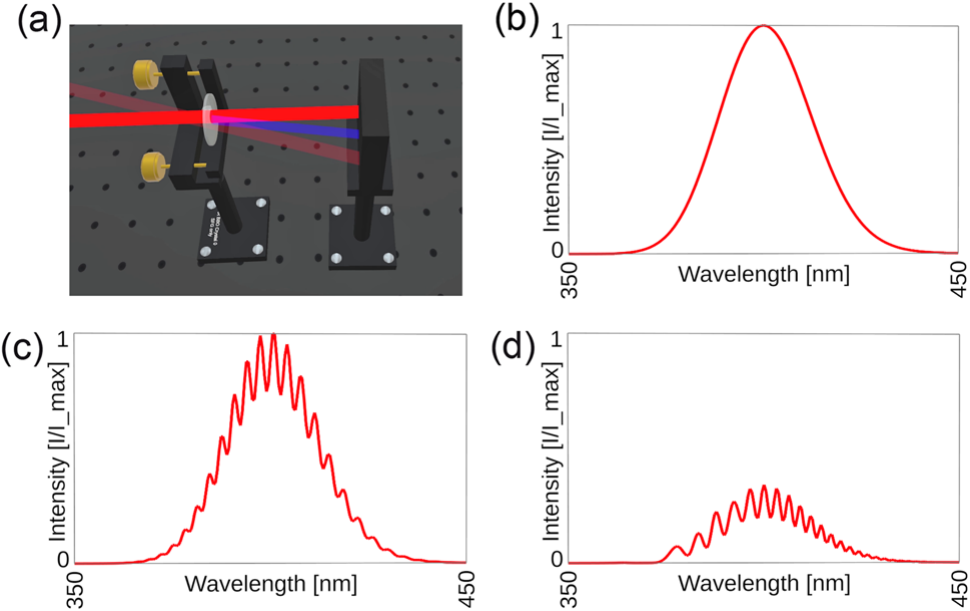
Sum-frequency generation. **a** Two fundamental beams of different power are incident from the left on a nonlinear BBO crystal. The transmitted fundamentals and the SFG emerge on the right. **b** Normalized SFG spectrum. **c** SFG spectrum in case of two collinear beams at an interpulse delay of 125 fs. **d** SFG spectrum (normalized to **c**), resulting from placing a dispersive optic with GDD $= - 200$ fs^2^ into one of the interferometer arms. The signal intensity decreases and features non-uniform fringe spacing due to chirp. All spectra were taken as screenshots from femtoPro

A femtoPro screenshot of SFG is provided in Fig. 3a. In this particular case, the two fundamental beams (red) are not overlapped perfectly in space in the BBO crystal and thus the SFG beam (blue) emerges only in the area of common overlap with a cross section that is smaller than the cross sections of either beam individually. The direction of the SFG beam is given by phase matching and occurs in between the two fundamentals as expected from Eq. (16). The respective SFG spectrum, recorded by a spectrometer in VR, is shown in Fig. 3b. In case of collinear overlapping beams, interference fringes become visible near maximum temporal overlap (shown in Fig. 3c for an interpulse delay of 125 fs). In this configuration, placing a dispersive optic into one of the interferometer arms leads to non-uniform fringe spacing and lower SFG signal intensity, which shows that chirp is realistically captured.

## Implementation

### Data structures

femtoPro was developed on the Unity game-engine platform [66] using the C# language. For reducing computational cost, time-consuming transformations are carried out in an automated fashion only when required. For example, pulse detection (Sect. 2.3) and pulse modification by linear response (Sect. 2.4) are carried out in the frequency domain, whereas second-order response (Sect. 2.5) is calculated in the time domain. The data structure for laser pulses contains entries for both the complex frequency envelope $\tilde{E}_\omega (j)$ and the complex time envelope $\tilde{E}_t(j)$ that are related via Fourier transformation. We do not know a-priori, however, in which order the laser beam will interact with various GOEs, sometimes requiring the spectral and sometimes the temporal field representation. Thus, to reduce the computational cost of Fourier-transforming at each optical element, we introduce an internal Boolean flag that determines whether the current spectral envelope entry reflects the laser pulse properties at the current position. If the spectral envelope is accessed and the Boolean flag is false, then the spectral envelope is first updated via Fourier transformation from the temporal envelope (but only then), and the flag is adjusted. The temporal envelope is treated analogously.

Each GOE is defined by a range of parameters for the interface and volume material properties as well as the geometry (thickness, open aperture, curvatures of interfaces). Since optical elements are typically not modified once they are created (except, e.g., the aperture radius for irises), all time-consuming calculations (such as the response functions) are carried out once when the GOE is created with the results stored numerically. This approach makes it possible to realize real-time treatment of linear and nonlinear optics including beam propagation and beam rendering.

### Physics simulation

All relevant steps of the physics simulation are performed when one or several laser beams hit a GOE. In that case, we call a subroutine (“method” in C#) with input parameters that contain a list of all incident pulses and the currently hit GOE. The method then performs the optical transformation and delivers as an output a list containing all transmitted, reflected, and newly generated nonlinear signal beams. These output pulses are then passed on to a beam-propagation logic for further tracing through space as described in Sect. 3.3.

The pulse-transformation algorithm proceeds in the following order:

1. Find the propagation vector of each incident beam as the vectorial difference between the points of intersection of the laser with the previous and with the current GOE’s active center plane.
2. Apply free-space spatial transformation [Sect. 2.1, Eq. (3)].
3. Create sum-frequency and second-harmonic pulses if applicable [Sect. 2.5, Eq. (16)].
4. Modify the pulse energy of incident pulses to take into account energy losses from generating nonlinear signals to fulfill overall energy conservation.
5. Initiate reflected and transmitted pulses as identical copies of each input pulse (with energies modified in step 4).
6. Apply geometric beam clipping due to finite-size open aperture.
7. Apply new beam direction due to focal length [Sect. 2.2, Eq. (6)].
8. Reflect direction for reflected beams.
9. Change complex radius of curvature due to focal length [Sect. 2.2, Eq. (7)].
10. Apply linear response separately to reflected and transmitted pulses with their respective response functions [Sect. 2.4, Eq. (12)].

Note that steps 6–10 are performed on all pulses, i.e., the linearly reflected and transmitted pulses as well as the nonlinearly created new beams. We consider “thin” optics for the spatial transformation and for the nonlinearities, i.e., the incident fields are assumed to generate the complete response uniformly throughout the material. We do not take into account that the incident fields are modified while propagating and thus may acquire dispersion, for example, that would lead to a varied response further along the propagation path within the same sample. Such effects have been investigated theoretically and experimentally in the literature [53, 67–70]. We omit them here for performance reasons. We do take into account dispersion, however, “summarily” on all pulses after the nonlinear signal generation process is complete, and thus dispersion is applied to the original pulses as well as to nonlinear-optics-generated new pulses once created.

### Beam-propagation logic

The physics simulation in Sect. 3.2 expects a list of input pulses and provides a corresponding list of output pulses with all relevant spatial and time–frequency pulse parameters set appropriately. To obtain the complete lists of incident pulses for each GOE, laser beams are traced through space. Interacting objects are detected even if only the edge of the beam, and not the center, intersects. Potential collisions are evaluated with active GOEs but also with other objects. For example, a laser beam might hit a mirror holder or lens holder to which the GOE is attached, a beam blocker, an “alignment card” held by the player, the hands of the player, or any other material object present in the scene. Further, we evaluate intersections with “virtual” planes that may be relevant for the mission objective. For example, we want to teach the player that no laser beam should leave the area of the laser table for safety reasons, and thus we check if any beam hits an appropriately defined virtual boundary.

The beam-propagation logic further needs to take into account that in the elements of linear optics, all beams can be calculated independently, and thus it does not matter whether the pulse-transformation method in Sect. 3.2 is called several times, i.e., individually for each incident beam, or once with a complete list of incident beams. For nonlinear optics, however, we need to provide a list of all incident beams at the same time. This brings out the complication that not all beams may be “known” yet because they, in turn, might arise from another GOE that itself needs a “complete” list of incident beams, and so on. Thus, we find a self-consistent solution iteratively, with a suitable break criterion to avoid potential infinite loops (such as in partially transmissive cavities).

**Fig. 4:**
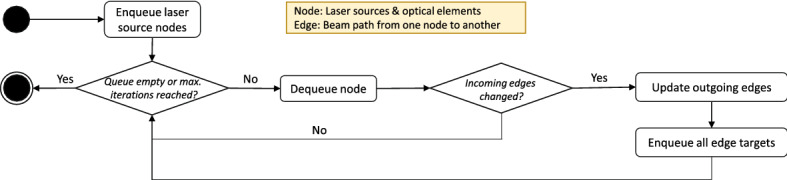
Logical flow of laser-path solver

The logical flow of the iterative laser-path solver algorithm is shown in Fig. 4. A formal graph contains nodes for all GOEs and other objects on the laser table. Then all possible sources of new laser beam elements (i.e., laser emitters themselves or optical elements from which a transmitted or reflected beam might arise) are added to a queue. Then, for each element of the queue, the outgoing pulses are calculated and it is checked whether this leads to modifications at other elements (“nodes”). If so, they are added to the queue such that they can be re-evaluated based on all additional beams that are incident on the element. Once the queue is empty, the formal graph has converged, and each GOE has been evaluated with the complete list of incident pulses in a systematic, self-consistent manner.

### Laser-beam rendering

**Fig. 5:**
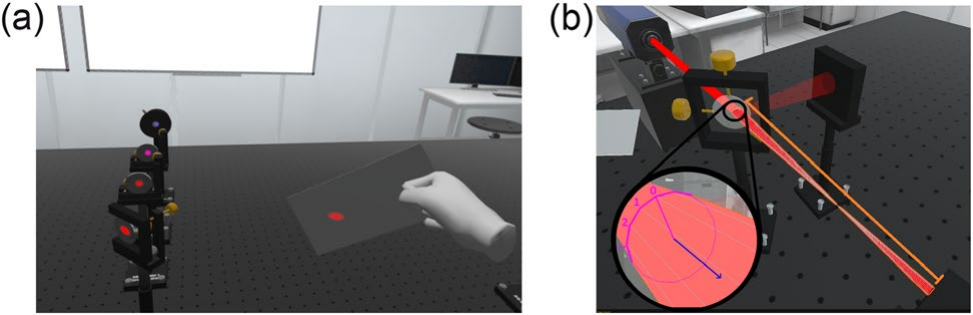
Laser rendering. **a** Cross-section visualization on an alignment card as in a real lab. **b** Full beam visualization. This scene shows a laser beam hitting a biconvex lens placed at an angle that leads to a focused transmitted beam and to a divergent beam reflected off the first surface. For the transmitted beam, the polygon mesh is superposed (see also inset). The propagation distance is indicated in orange

Real-world laser beams are invisible unless they hit the eye (or a detector) directly. Partial visibility can be achieved by employing scattering media in the beam path, such as fog. This is not practical in real ultrafast laser labs because of eye-safety concerns. Even weakly scattered fs light pulses may still have high enough intensity to cause eye damage. Additionally, using fog is detrimental to sensitive optical components and the laser function itself that typically demand low humidity. Thus, in practice, ultrafast laser lab practitioners use “alignment cards,” typically of the size of conventional business cards, to “follow” laser beams through space and ensure their correct path. Thus the laser beam is visualized indirectly by its beam cross section scattered off the alignment card. Again, for safety reasons, one has to ensure that the scattered light is not dangerous. In the case of infrared light, one may use specially infrared-sensitized fluorescent cards or infrared-sensitized cameras. We provide such a realistic simulation as one optional mode in which only the cross section of a laser beam is rendered when it hits an object, with the luminance adjusted to the actual intensity of the laser beam (Fig. 5a).

As a second option, we exploit that a VR environment provides new opportunities for the didactic visualization of laser beams not available in the real world, and we render a visible beam path in space approximating the Gaussian beam-propagation physics. In principle, we need to plot circles of a radius given by Eq. (2) for each position *z* along the propagation length. In computer graphics, objects are represented by polygon meshes with straight edges, which is also how we approximate the Gaussian beams as shown in Fig. 5b.

Note that the visualization is completely independent of the physics simulation of Sects. 2 and  3.2, such that any precision compromises implemented for speeding up the rendering do not influence the accuracy of the physics model.

The color used for rendering the laser beam mesh and the beam cross section upon scattering are calculated by assuming a “standard observer” defined by the “Commission Internationale de l’Éclairage” (CIE) in its most recent implementation [71] and taking into account the given spectrum of the fs pulse.

### VR scene and VR interactions

We have set up a laboratory scene containing an optical table on which GOEs can be placed. Players can move around the table either by walking in real space or by a teleportation function using the VR controller. All GOEs are defined as interactable elements. When the player’s virtual hand position is close to a GOE, it is highlighted to indicate that it is ready for interaction. Using the “grab” button, the GOE can then be picked up and placed elsewhere on the table. Separate from this coarse positioning, the player can use the “trigger” button to adjust the height and coarse angle of the GOE by simultaneously moving the hand up or down and by performing a rotation gesture, respectively.

**Fig. 6:**
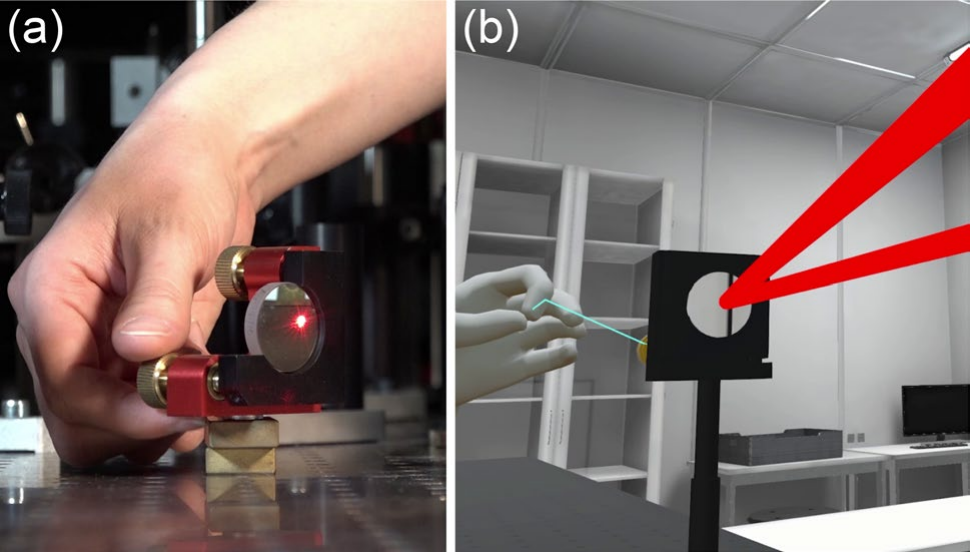
Mirror alignment. **a** The interaction with real fine-adjustment screws using a rotational motion with fingers and hand is translated into **b** VR by implementing a hand gesture similar to turning a crank

We placed particular emphasis on an angular fine-tuning procedure that resembles the real-world turning of fine-adjustments screws of typical mirror holders (Fig. 6a). In femtoPro, using the trigger button, players can interact with a horizontal or vertical fine-adjustment screw and perform a rotational motion with their hands, similar to turning a crank handle. This turns the VR screws and correspondingly adjusts the length of the screw and thus the tilting angles. The helper lines (cyan) provide visual feedback to the user even when the hand is moved away from the initial interaction point with the screw (Fig. 6b). In addition, haptic feedback (i.e., a slight vibration) is provided via the VR controllers while the screw is turned. The similarity in practical procedures between real world and VR allows femtoPro to be used not just for generic optics education, but to train specific and transferable alignment skills.

A second type of optics holder is provided for elements whose vertical and horizontal position need to be fine-tuned, rather than their angle. In that case, two fine-adjustment screws are implemented for vertical and horizontal displacement to allow precise alignment of the laser beam onto the center of the GOE such as for lenses (see, e.g., Fig. 5b).

Currently, the following GOEs are implemented in femtoPro: flat and curved mirrors, transmissive lenses, beam blockers, beam splitters, iris apertures (that can be opened and closed again by an interactive half-rotational gesture), neutral-density filters, spectral filters, spectrometers, and power meters. We have also realized mechanical delay stages on which GOEs can be placed (in particular mirrors) to realize time delays.

A virtual laptop computer contains data-acquisition software that controls the positions of delay stages, reads out power meters and spectrometers and displays the results on the screen. In this fashion, time-resolved experiments can be simulated.

## Usage

### Example scene

**Fig. 7:**
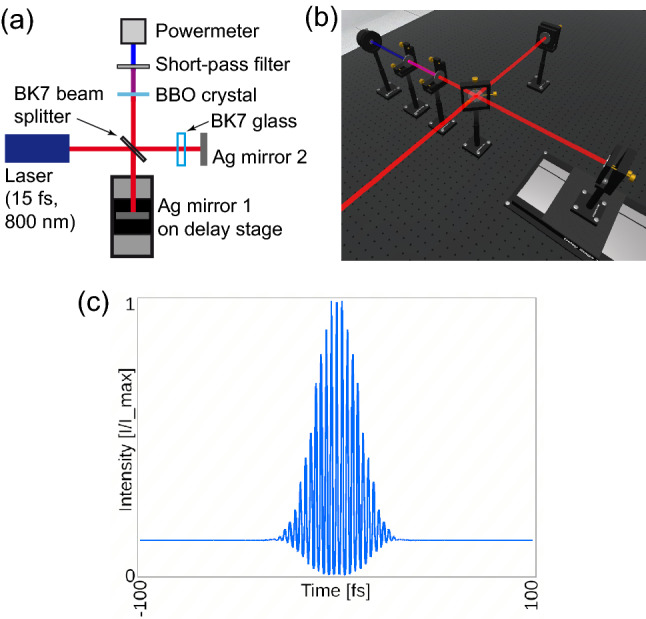
Interferometric autocorrelation. **a** Schematic layout. **b** Screenshot from femtoPro of a part of the setup using the option of visualized beams. **c** Screenshot from femtoPro of simulated measurement data as a function of time delay

As an example, we have set up a Michelson interferometer with second-harmonic generation. The schematic layout is shown in Fig. 7a. We have then defined the ideal positions and angles of all GOEs such that optimal collinear beam overlap is reached, and we have realized these positions by coding the desired positional values numerically into a femtoPro scene, rather than placing GOEs by hand in VR. This was done in order to demonstrate the simulation under ideal circumstances.

The screenshot in Fig. 7b shows a part of the setup. The beam splitter recombines two time-delayed fundamental beams (red) collinearly and steers them onto a nonlinear crystal for second-harmonic generation. The frequency-doubled light and the remaining fundamental both emerge from the crystal (violet). A short-pass filter transmits only the high-frequency components (blue) that are then steered towards a detector. In the case of non-collinear second-harmonic generation, more than one beam would emerge from the crystal.

The user can interact with the virtual laptop contained in the scene to control the delay stage via a data-acquisition interface that allows the setting and scanning of delay positions. The result of such a measurement is shown in Fig. 7c displaying the well-known interference fringes and 8:1 peak-to-background contrast ratio expected of an interferometric autocorrelation [9]. Note that this is no “direct” simulation using a correlation formula that is conventionally employed in such a context [9]. The results are rather obtained from explicit simulation of the physical (Gaussian) propagation of all beams through space including interaction with the various GOEs, and from moving the delay stage to varying positions.

**Fig. 8:**
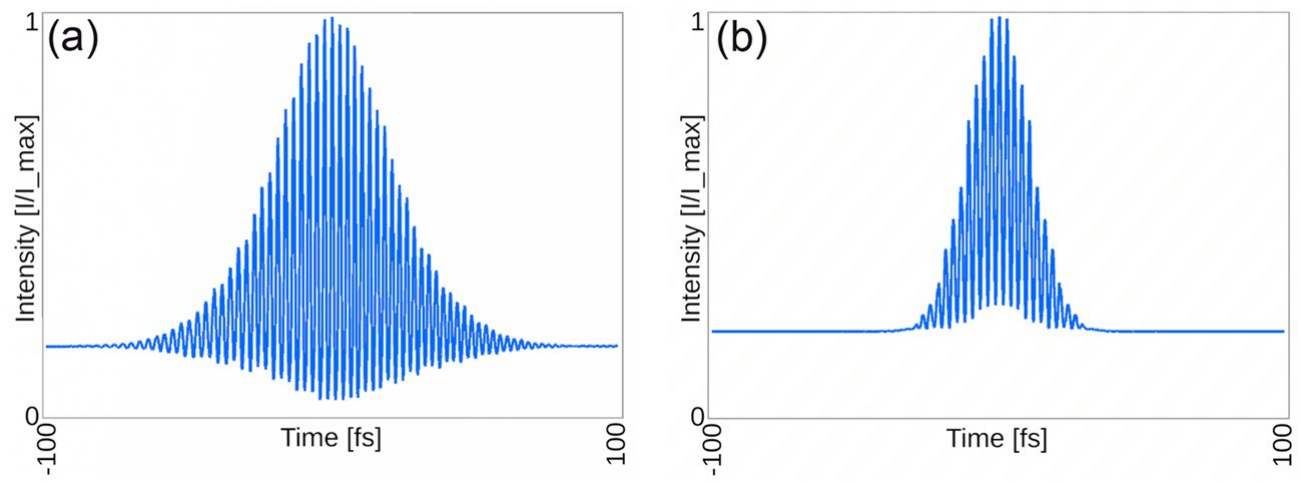
Correlation measurements obtained by modifying the setup of Fig. 7. **a** Cross-correlation with a 1.5 mm plate of BK7 glass inserted in one of the interferometer arms. **b** Autocorrelation after misaligning one of the interferometer arms. All autocorrelation plots were taken as screenshots from femtoPro

For illustration, we modified the setup of Fig. 7 by placing a 1.5 mm plate of BK7 glass (anti-reflection coated) in one of the interferometer arms. The resulting dispersion from passing the plate twice results in temporal broadening. Thus the interferometer is no longer balanced, and the resulting cross-correlation in Fig. 8a reveals the increased pulse duration.

As a second modification example, we removed the BK7 glass plate and misaligned mirror 1 in Fig. 7a by a lateral angle of 0.002$^{\circ }$. The resulting autocorrelation in Fig. 8b reflects the imperfect beam overlap in the form of an intensity profile deviating from the ideal 8:1 peak-to-background contrast ratio, which is in line with the real-world observation.

In addition to spectrally integrated signals, one can also record spectrally resolved signals by evaluating Eq. (10). In that case, it is possible to simulate frequency-resolved optical gating (FROG) measurements [10] or, in the case without employing nonlinear crystals, spectral interference signals [57].

### Training missions

The example in Sect. 4.1 has illustrated the capabilities of the physics simulation. For teaching purposes, we have developed a training mission system in which the user is guided, step by step, through an alignment procedure to realize a specific optical setup and perform prescribed VR experiments. One mission consists of individual tasks that have to be performed, and each task is checked for completion via predefined conditions, such as whether a laser beam hits a certain GOE. Once the condition is fulfilled, the task is marked as completed and the user may proceed to the next step.

We have implemented a number of training missions, and more can be defined according to didactic needs. Example missions contain step-by-step instructions on how to fix the position and direction of a beam using a set of two irises or how to align a telescope. More advanced missions teach users how to align a delay stage or how to set up an interferometer and perform an autocorrelation measurement.

### Application scenarios

The core application entails participating in virtual exercises and performing virtual experiments. Both exercises for students and experiments for scientists revolve around building laser setups and performing measurements in these virtual setups. In extension, users further want to practice eye safety and the handling of equipment. To this end, users want realistically simulated interactions and a safe way to learn how to avoid potentially dangerous actions. These core functionalities are provided by femtoPro, and several extensions provide additional application scenarios as summarized below.

#### Academic teaching

femtoPro can be used to set up a complete one-semester practical optics lab class at a fraction of the cost for a real lab class, which leads to significant financial savings for academic departments. In addition, the immediate feedback provided while using femtoPro allows a much more effective training than with unsupervised exercise and thus to an increase in quality. Supervision can be realized with much reduced personal supervision due to the automated training missions. femtoPro is also useful to train new members of scientific working groups in the basic procedures required in the laser lab and thus provides a consistent practical introduction to the topic.

#### Training of optics professionals

Optics companies such as manufacturers of lasers and related optical or optomechanical components have to provide practical training to their new staff. For example, service engineers need to be able to set up, repair, and align complex optics setups at the site of customers. Thus they have to acquire optical alignment skills and practice safe laser handling which could be provided by femtoPro. There is also the potential to include specialized missions for specific setups.

#### Remote support

Supervised remote teaching will become possible by implementing an additional “multi-player” mode in which several persons can log into the same VR lab and chat via an audio channel. Possibilities for such groups are a teacher and one or several students, but also an optics expert and an optics customer. In this way, one person can demonstrate particular skills and handling schemes to others. This is helpful in terms of limited availability of laboratory access for large numbers of students (or customers). But also considering the cost of travel, training over a distance has significant advantages.

#### Laser safety classes

Employers using lasers of classes 3R, 3B, and 4 generally have to appoint laser safety officers. These have to attend training classes, pass an exam, and have to participate in refresher seminars in subsequent years. Relevant lasers are used not only in the scientific and engineering context, but also in many areas of material processing and industrial fabrication, all the way up to medical applications and consumer electronics such as laser light shows. In all these cases, laser safety officers with certificate are required by law. femtoPro could be used as a supplement to such classes, provide practical training thus enhancing the safety aspects and unifying the teaching of safe working procedures.

#### Planning and testing of optical setups

Planning optical setups is usually done on the drawing board and some parts may be simulated by ray-tracing or other optics simulation software. However, this does not provide a realistic experience for the challenges that will arise with an actual alignment. femtoPro can be used to design, test-build, and simulate new experiments. The VR hands-on real-time feedback provides a handle for evaluating how practical an actual setup will be in the real world and saves resources in prototyping procedures.

#### Supplementary information for publications

Scientific publications with new designs of optical setups typically contain a two-dimensional sketch of the optical components, in most cases not drawn to scale. This makes it challenging for other researchers to reproduce the design, and in particular to reproduce the often sensitive alignment. As a consequence, the spreading of new developments may be slowed down, hindering scientific progress. femtoPro could be used as a complementary part of scientific optical publishing. For example, the authors could define a suitable femtoPro scene with an appropriate descriptive language that contains the specifications and positions of all optical components as in their real work. Furthermore, the authors could define the alignment routine as a “training mission.” Then, any other scientist (either from different groups or even other members from the same research group) could follow, step by step, the published setup and alignment procedures, which would enable them to follow the same steps also when setting up the real experiment.

#### Education of high-school students

Physics education in high schools rarely provides the opportunity for students to obtain practical experience because of limited financial and personnel resources. femtoPro could offer practical experience to high-school students in an engaging and playful manner, yet with correct scientific contents. For example, teaching optics and the manipulation of light beams with lenses and mirrors forms a standard part of high-school curricula. Using femtoPro, students could experiment with lenses of different focal length and immediately see how this affects the beam transformation. Thus it is possible to construct and understand the working principles of telescopes or other optical components. This would provide a complementary practical part of education. High schools could set up such a VR optical laboratory at a fraction of the cost of a real laboratory and avoid liabilities arising from potential eye-safety hazards with minors.

#### Outreach activities for the general public

Lack of scientific knowledge is detrimental for society as a whole and leads to strong economic and social problems. Raising the awareness for the power and predictive accuracy of natural sciences can help to diminish such problems in the long run. femtoPro could play a part by introducing the public to scientific questions and in particular to concepts of optics. This topic is well suited for outreach activities because everyone has some experience with light and optics from various types of consumer electronics, lighting, eye goggles, supermarket cashier scanners, laser printers, etc., to which they can relate. We have successfully used femtoPro in an outreach activity at the general-public science fair “Highlights of Physics” at Würzburg [72], where visitors could wear a VR headset and play an exemplary femtoPro training mission.

## Conclusion

Lasers are used in many disciplines of science, engineering, manufacturing, medicine, and daily life. Safe handling of lasers is a common requirement across all applications. Our virtual-reality (VR) ultrafast laser laboratory simulator “femtoPro” provides an eye-safe environment in which users can learn operating procedures in a practical manner. Step-by-step tutorials are combined with automated checks if individual tasks have been realized. This allows the training of alignment procedures even of complex optical setups.

Spatial properties of laser beams are simulated using Gaussian beam propagation, and spectral–temporal aspects of femtosecond pulses are simulated using response functions. Thus, nonlinear phenomena can be simulated. At present, second-order non-resonant processes are included allowing the treatment of sum-frequency and second-harmonic generation. Third-order response is currently under development and would allow real-time simulation and training of common time-resolved spectroscopy experiments like transient absorption or coherent two-dimensional spectroscopy.

In developing a physical model for the simulation, we have chosen a level of approximation such that features essential for didactic learning purposes are taken into account. On the other hand, we have included only such levels of detail that real-time simulations of all effects are feasible. Here, “real time” has the meaning that a user has the same visual experience of immediate feedback during alignment as in a real-world laser lab. This typically requires >50 frames/s VR image update rates.

In the present work, we have described the essential features and application scenarios of the laser lab simulator and provided several specific examples of scenes and applications. We hope that femtoPro will open new possibilities for practical optics teaching and training, including remote interactions, and thus contribute to avoiding accidents. VR applications are an intriguing component of digital teaching innovations that are currently in high demand, and we trust that femtoPro can fulfill such a role for the optics community.

## Data Availability

Data underlying the results presented in this paper are not publicly available at this time but may be obtained from the authors upon reasonable request.
